# Molten Pressure of Polyvinyl Chloride Under Processing Parameters in Counter-Rotating Twin-Screw Extrusion

**DOI:** 10.3390/polym17212809

**Published:** 2025-10-22

**Authors:** Jen-Sheng Weng, Hsi-Hsun Tsai, Jia-Wei Liu

**Affiliations:** 1 Doctoral Program in Industrial Management, National Yunlin University of Science and Technology, Yunlin County, Douliu 64002, Taiwan; d11262004@yuntech.edu.tw; 2 Plastics 3rd Division, Nan Ya Plastics Corporation, Chiayi County, Taibao City 612005, Taiwan; 3 Research Center for Intelligent Medical Devices, Ming Chi University of Technology, New Taipei City 24301, Taiwan; 4 Department of Mechanical Engineering, Ming Chi University of Technology, New Taipei City 24301, Taiwan

**Keywords:** extrusion, thermal analysis, screw, polyvinyl chloride, twin-screw extruder, pressure distribution

## Abstract

Thermal properties significantly affect extrusion energy efficiency and polymer processing. Relevant parameters include melt temperature, viscosity, and specific heat impact energy consumption, while thermal degradation limits processing temperatures within the screw and barrel. Traditional empirical methods used in polymer extrusion are often hindered by the complex relationship between screw speed and energy efficiency. Numerical simulations, particularly those using ANSYS Polyflow, offer a more precise approach for visualizing temperature, pressure, and shear rate distributions in the molten polymer, enabling better control of extrusion conditions. The screw’s geometric configuration, which includes sections for conveying, compressing, kneading, and mixing, plays a key role in determining flow behavior and performance. Studies on polymers using various screw configurations have revealed that screw designs with lower compression ratios enhance throughput and reduce melt temperature. Additionally, barrier screw designs improve the polymer melting efficiency. In this study, ANSYS Polyflow simulations were applied to analyze the flow behavior of molten PVC in a counter-rotating twin-screw extruder, focusing on the effects of screw speed and inlet flow rate on pressure, temperature, and velocity distributions. The results indicated optimal extrusion conditions for preventing degradation, with an ideal outlet rate of 439 kg/h at a screw rotational speed of 43 rpm. The pumping pressure of molten PVC by a twin-screw approach would be enough for entering the extrusion die.

## 1. Introduction

The thermal properties of polymers play a critical role in extrusion energy efficiency and processing characteristics. Parameters such as melt temperature, thermal conductivity, viscosity, and specific heat influence energy consumption during extrusion, while thermal degradation limits the maximum processing temperature within the barrel and screw [1,2]. Traditionally, an empirical approach based on trial and error has been used in polymer extrusion. However, the nonlinear and often counterintuitive relationship between energy efficiency and screw speed can complicate the process of setting extrusion conditions due to the combined effects of temperature, rheology, and wall slip [3]. Numerical methods offer a more reliable means of visualizing the temperature, pressure, and shear rate of molten polymer within the barrel and screw, enabling extrusion conditions to be set more accurately. The geometric characteristics of the screw are crucial in these simulations, as they determine the physical properties of the molten polymer during extrusion [4]. A screw typically consists of various configurations for conveying, compressing, kneading, and mixing [5]. The fractal nature of each configuration plays a significant role in understanding the mechanical behavior and performance of the screw across different applications [6].

Using rapid cooling techniques, the extrusion behaviors of polystyrene (PS) and low-density polyethylene (LDPE) were studied using five screw configurations: a general-purpose screw, a barrier screw, and three composites with different fractal geometries. The general-purpose screw, featuring a square-pitch design, had equal-length feed, transition, and metering sections and a compression ratio of 2.0, lower than the typical industrial values of 2.8 for LDPE and 3.0 for PS. A lower compression ratio allows for higher throughput per screw revolution, thereby reducing the increase in melt temperature. The barrier screw design incorporates a dedicated melt channel to promote complete polymer melting before the metering section, improving extrusion efficiency and ensuring consistent product quality [7]. Screw pitch and flight geometry significantly influence pumping capacity and channel fill in twin-screw extrusion, directly impacting torque requirements. Shallower grooves promote pressure build-up and increase shear rates, resulting in elevated torque, while deeper grooves enlarge channel volume and reduce shear under equivalent shaft speeds [8,9]. Both experimental studies and computational fluid dynamics (CFD) simulations have demonstrated that groove depth alters the shear and pressure fields, influencing mixing efficiency and torque. Flight depth governs self-cleaning dynamics, axial mixing intensity, and local shear rates. Distinct configurations exhibit varying shear and pressure characteristics, some favoring high torque due to intensified shear, others enabling superior pressure development at comparable speeds [9]. The groove-to-flight ratio is thus a critical design parameter for optimizing the trade-off between mixing performance and torque consumption.

Kneading blocks and mixing elements further affect torque through their roles in dispersive and distributive mixing. Element width and stagger angle determine the balance of these mechanisms. Wider kneading blocks intensify elongational and shear deformation, increasing torque demand, whereas narrower distributive elements divide the flow with reduced extensional work [10]. Thread profile geometry also influences torque via its effect on local shear rates. Triangular and fileted thread profiles minimize dead zones and enhance shear, improving dispersion but increasing torque relative to low-shear designs [11,12]. Adjustments to end-face geometry affect local shear gradients and viscosity fields, with simulation studies revealing significant torque variations based on specific end-face configurations [13]. Reverse elements and flow restrictions raise pressure and enhance mixing but also substantially increase shaft torque and specific mechanical energy (SME) by restricting forward flow and increasing local fill [8,14]. Their use must align with torque capacity and process objectives.

Thermal conditions critically influence torque via melt viscosity. Increasing barrel and die temperatures (e.g., from 100 °C to 140 °C) decreases apparent viscosity, die pressure, and torque, thereby reducing SME [15]. Although the viscosity-temperature relationship follows standard rheological models, non-uniform temperature profiles cause spatial variation in torque. Screw speed exhibits a nonlinear relationship with torque. Higher speeds raise shear and viscous dissipation but may reduce residence time and alter morphology, particularly in polymer blends [16,17]. Feed rate influences torque through fill level, with starved conditions reducing and full fill increasing torque. Lastly, material rheology, particularly with fillers or fibers, affects torque and wear. Configuration-specific studies confirm strong dependencies between torque, SME, and material composition in systems such as biomass or fiber-filled polymers [18].

The mixing performances of various screw configurations have been further investigated using color-coded polymers and interrupted extrusion experiments [19]. Wilczyński et al. [20] conducted experimental research on solid conveying in counter-rotating twin-screws, revealing that solid particles are initially transported above the screw near the hopper but mostly fall to the bottom of the barrel downstream, where they are conveyed along the sleeve. Only a small fraction remains above the screw, indicating a non-uniform heating distribution across the screw cross-section, despite rotation facilitating thermal exchange by drawing heated particles into the main screw channel. In their review of screw mixing, Wilczyński et al. highlighted that molten properties in each screw section are influenced by the preceding section, emphasizing the need for an optimized screw design to ensure efficient mixing and consistent extrusion quality.

Several studies have examined the co- and counter-rotating twin-screw extrusion processes [21,22,23]. Non-isothermal flow simulations in co- and counter-rotating twin-screw extruders using ANSYS Polyflow (Canonsburg, PA, USA) have provided three-dimensional transient thermal results, which were validated against experimental data, demonstrating this method’s potential to optimize extrusion configurations for specific processing requirements [24]. The flow behavior and pumping characteristics of polymer melts in counter-rotating twin-screw mixers were evaluated through ANSYS Polyflow simulations [25]. For helical screw elements, increasing throughput was found to reduce the pressure gradient, ultimately leading to negative values. In contrast, mixing elements exhibited increasingly negative pressure gradients as throughput increased [26].

Polyvinyl chloride (PVC), due to its high viscosity, is ideal for extrusion in pipe production and continuous parts. However, the thermal sensitivity of PVC during extrusion makes it prone to degradation [27]. PVC injection molding has constrained temperature and shear rate owing to its temperature sensitivity and high viscosity [28]. The extrusion and compound of PVC have much more challenge due to its higher viscosity. In this study, to derive extrusion conditions that prevent degradation, ANSYS Polyflow is employed to systematically analyze melt flow behaviors during twin-screw compounding and die extrusion. Simulation results are then used to inform the design process, particularly focusing on molten temperatures in the metering section of the screw. A three-dimensional analysis of the screw/barrel system and extrusion die is conducted to ensure that degradation-free extrusion is performed and to optimize the extrusion conditions.

## 2. Materials and Methods

**Figure 1:**
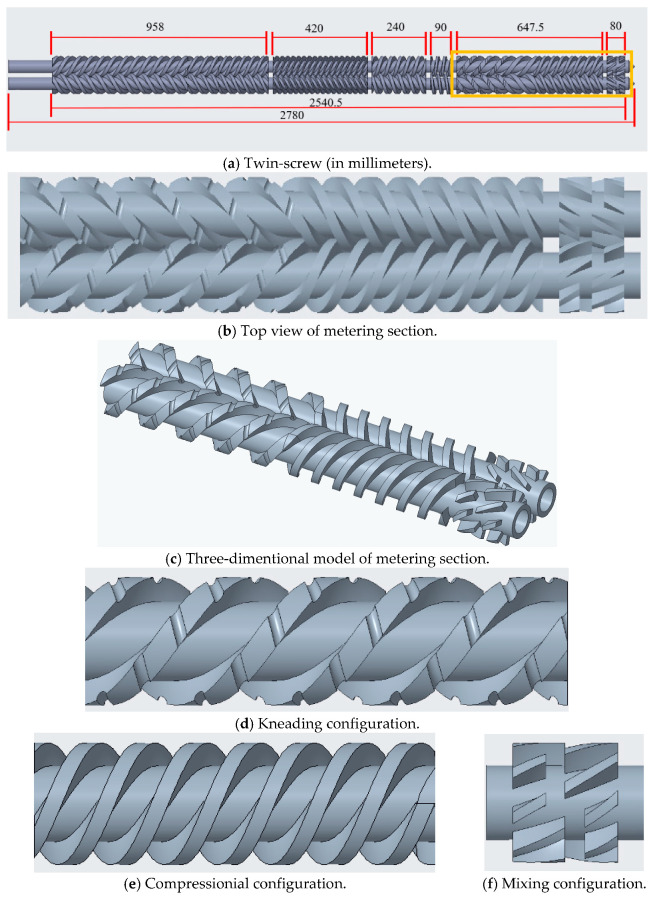
Counter-rotating twin-screw.

Three-dimensional modeling of the screw, barrel, and mold was performed using Creo Parametric 6.0.2.0 (Boston, MA) (https://www.ptc.com/en/products/creo/parametric, 20 October 2025). A geometric representation of the metering section of the counter-rotating twin-screw is shown in Figure 1. Subsequently, the melt flow domain was discretized using ANSYS 2024R1 (https://www.ansys.com/, 20 October 2025), and the ANSYS Polyflow module was employed to define the initial and boundary conditions before conducting a simulation analysis. The function of the currently used software is limited to analyzing fully molten materials, and, therefore, the metering section of the screw, as depicted in Figure 1, was considered for analysis. Figure 1b,c illustrate the metering section of the screw, which consists of three distinct configurations for kneading, compressing, and mixing. The lengths of these configurations are 345 mm, 302.5 mm, and 80 mm, respectively, with a gap of 20 mm between the compressing and mixing configurations. Figure 1d shows the kneading configuration, with a thread width of 22.2 mm, a height of 16.5 mm, a pitch of 210 mm, and three threads. Circular overflow grooves were incorporated into the threads to enhance the kneading effect; these grooves had diameters of 9 mm and depths of 4.5 mm, along with groove pitches of 35 mm. The molten volume in this kneading configuration was 1334.79 cm^3^ during compounding. The compressing configuration, as is shown in Figure 1e, was trapezoidal in shape with three threads. It had a lower base of 16.2 mm, an upper base of 13.2 mm, a height of 16.5 mm, and a pitch of 120 mm. The molten volume in this configuration was 1380.08 cm^3^. The mixing configuration, as is depicted in Figure 1f, was rectangular, with a width of 8 mm, a height of 16.5 mm, and a pitch of 392 mm. The molten volume in this mixing configuration was 737.34 cm^3^.

**Figure 2:**
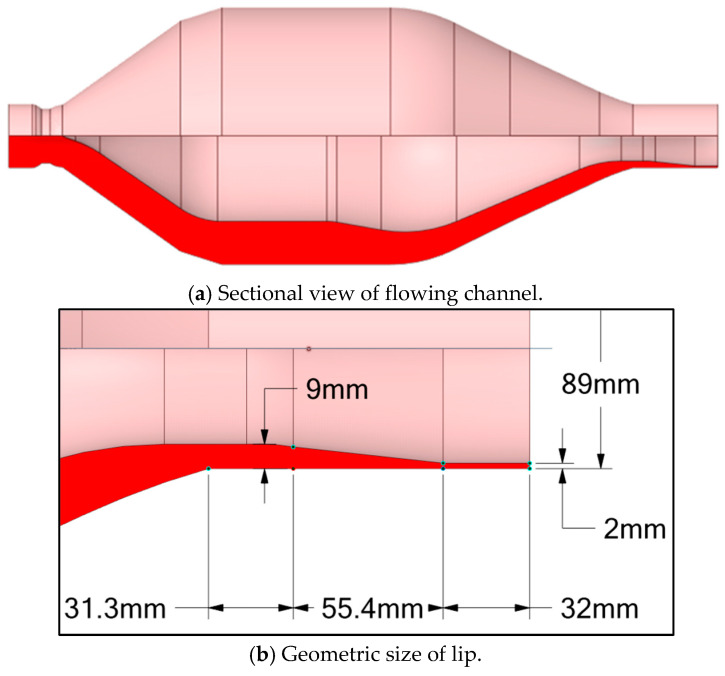
Extrusion die (in millimeters).

**Figure 3:**
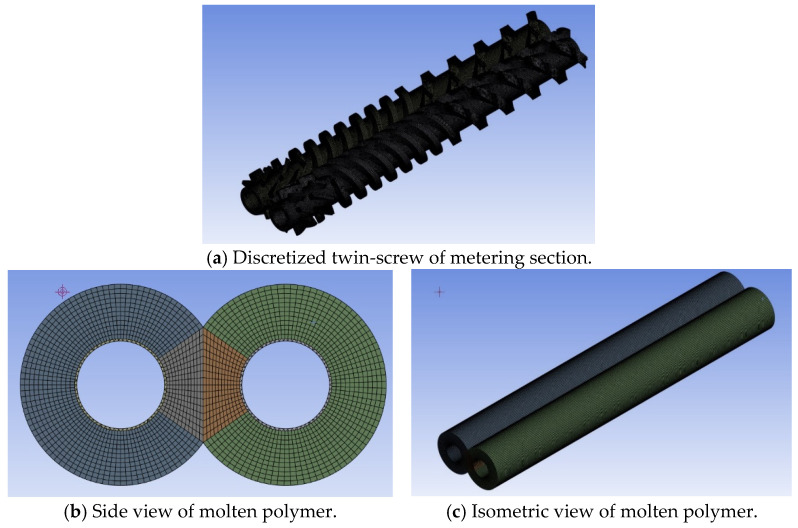
The discretized molten volume between the screw and barrel in the metering section.

Figure 2 illustrates the extrusion die for a PVC pipe, featuring an outer diameter of 89 mm and an inner diameter of 85 mm. The molten PVC within the metering section of the barrel/screw system has a volume of 7598.706 cm^3^. The mesh for this molten PVC consists of 4,967,819 elements and 1,932,614 nodes. Additionally, the molten PVC within the flow channel of the extrusion die has a volume of 26,689.6 cm^3^. After discretization, the mesh of the molten material within the extrusion die comprises 1,564,636 elements and 667,563 nodes, as shown in Figure 3.

**Figure 4:**
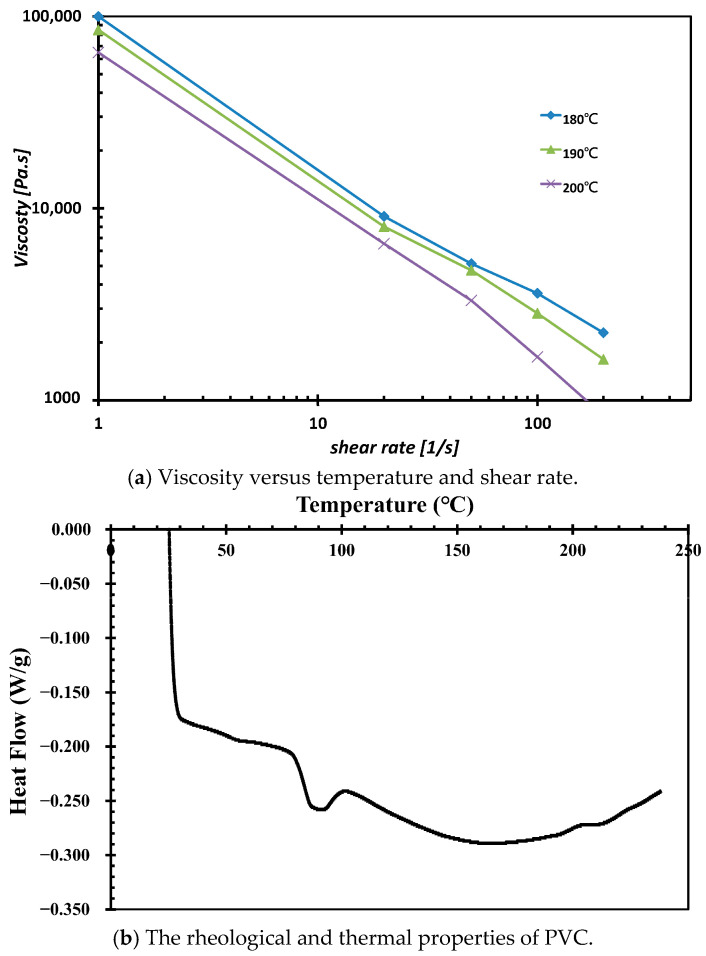
The rheological and thermal properties of PVC.

The viscosity of PVC at different shear rates was analyzed using GÖTTFERT’s rheograph, as shown in Figure 4a. In Figure 4b, PVC was characterized using a differential scanning calorimetry (DSC) instrument (TA Instruments Discovery DSC 25, Delaware, USA) under a nitrogen atmosphere. The sample was heated to 240 °C at a ramp rate of 10 °C/min. The results indicated a starting melt temperature of approximately 78 °C, with a peak melting temperature occurring at 88 °C. The PVC material properties used in the analysis are summarized in Table 1. The compounding conditions for both the extruder and die are outlined in Table 2, with the screw rotational speed set to 43 rpm and the inlet extrusion rate specified. The outlet was configured to have no additional normal or tangential velocities, and no-slip conditions were applied to the barrel wall, ensuring that the melt had no relative or tangential velocity at the barrel wall.

## 3. Results and Discussion

**Figure 5:**
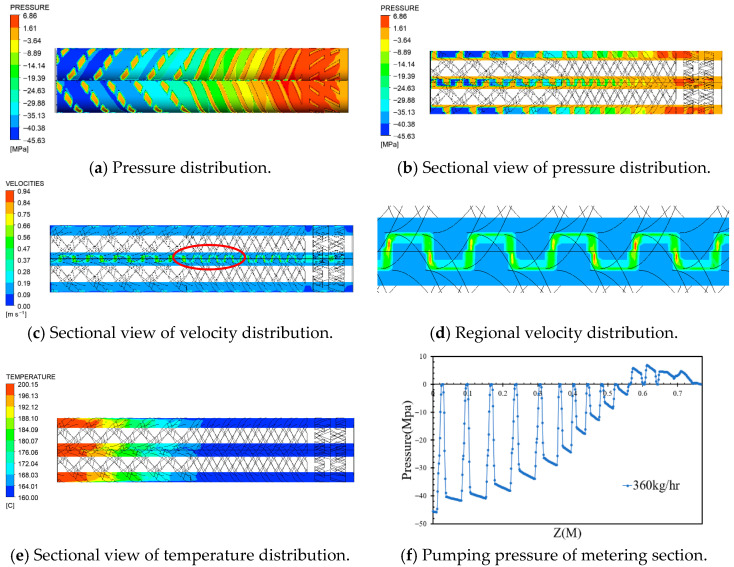
The compounding effects of a counter-rotating twin-screw (43 rpm, outlet 360 kg/h).

To investigate the influence of the screw rotation speed and inlet flow rate on the pressure, velocity, and temperature distributions of molten PVC within the metering section of the screw and barrel system, a series of simulations were conducted using ANSYS Polyflow 2024 R1. The barrel temperature was maintained at 200 °C in the kneading section of the metering zone and at 160 °C in the compressing and mixing sections. The twin-screw extruder operated at a counter-rotating speed of 43 rpm, while the inlet flow rate of molten PVC ranged from 360 to 500 kg/h. At an inlet rate of 360 kg/h and a screw speed of 43 rpm, the pumping pressure within the metering section was 45.63 MPa, as shown in Figure 5a,b. The outlet pressure at the screw/barrel interface was set to zero in the simulation to calculate the inlet pressure, with the absolute value representing the pumping pressure. The pumping pressures for the kneading and compressing sections were approximately 15 MPa and 30 MPa, respectively, while the pressure loss in the mixing section was 6.86 MPa. Figure 5c,d illustrate the velocity of the molten material in the inter-screw region, influenced by the counter-rotating motion. Stagnation zones were identified near the barrel wall, particularly around the mixing section. Figure 5e shows the temperature distribution, where the melt temperature decreases gradually from 200 °C to 160 °C from the kneading to the mixing configurations. Finally, Figure 5f presents the variation in molten pressure within the inter-screw region, showing pressure fluctuations in relation to the screw threads, as derived from the data presented in Figure 5b.

**Figure 6:**
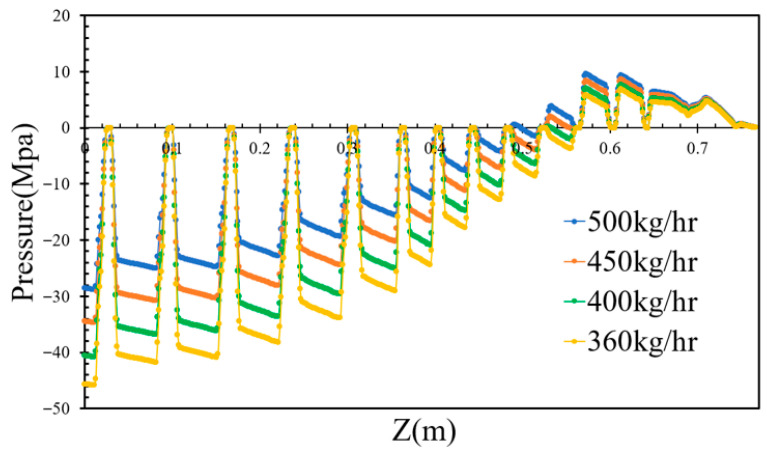
The pumping pressure with respect to the inlet rate.

**Figure 7:**
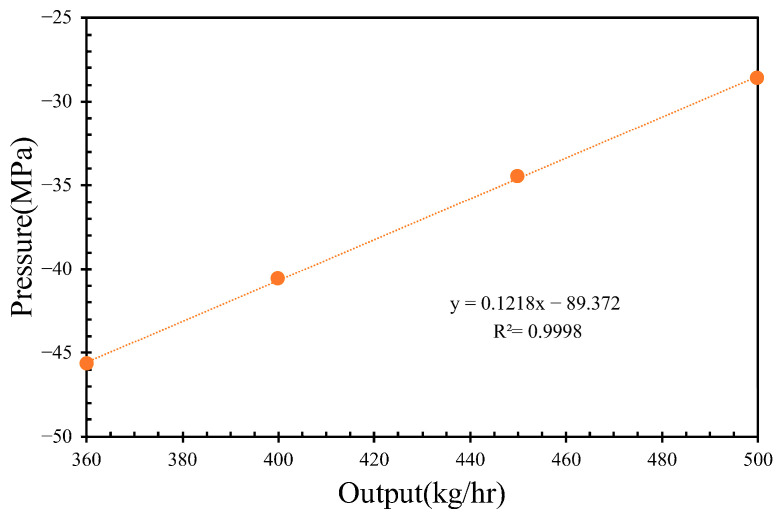
The inlet pressure with respect to outlet molten polymer.

**Figure 8:**
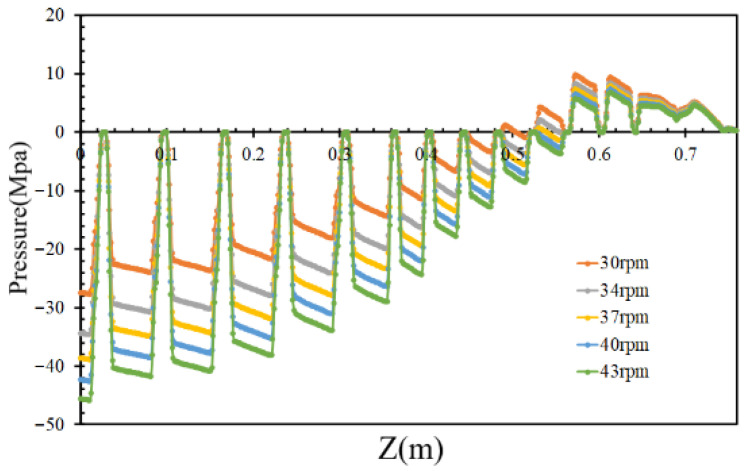
The pumping pressure with respect to the rotational speed.

**Figure 9:**
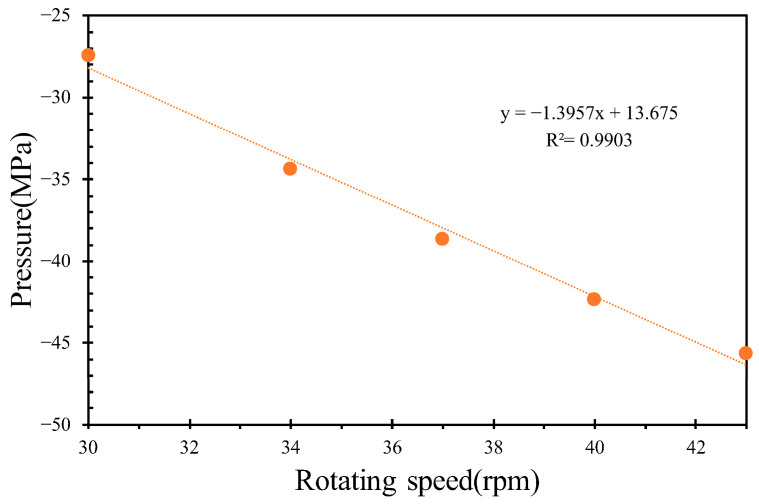
The inlet pressure with respect to the outlet rate.

By varying the inlet flow rates to 400, 450, and 500 kg/h while maintaining a constant screw rotational speed of 36 rpm, the pressures of the molten PVC in the inter-screw region, which typically fluctuate with respect to the screw threads, were derived, as shown in Figure 6. Additionally, the pressure loss in the mixing section slightly increased with respect to the inlet flow rate of molten PVC. As the outlet pressure was assumed to be zero, the pumping pressure decreased with an increase in the inlet flow rate. Figure 7 further demonstrates that, within the metering section, the pumping pressure was linearly proportional to the outlet flow rate of molten PVC at a screw rotational speed of 43 rpm. At an inlet rate of 360 kg/h, simulations were performed at screw rotational speeds of 43, 40, 37, 34, and 30 rpm. The results showed that the pumping pressure in the metering section increased at higher screw rotational speeds. As depicted in Figure 8, the pressures in the inter-screw region of the counter-rotating twin-screw also increased with respect to the screw rotational speed at the same inlet flow rate of 360 kg/h. The pressure loss in the mixing section exhibited a negative correlation with the screw rotational speed, indicating that a higher screw speed reduces the pressure loss. Furthermore, as shown in Figure 9, an increase in the screw rotational speed led to a corresponding increase in the pumping pressure within the metering section.

**Figure 10:**
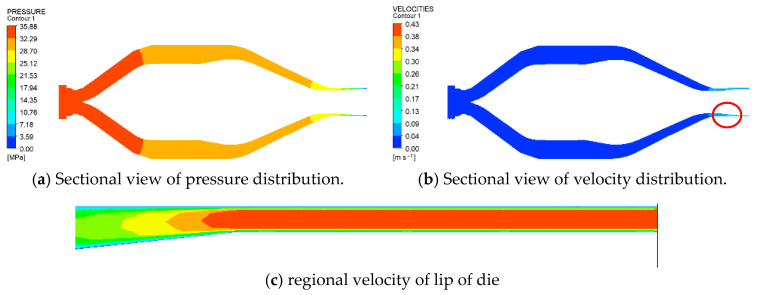
The extrusion effects of molten PVC within the die (43 rpm, outlet 500 kg/h).

**Figure 11:**
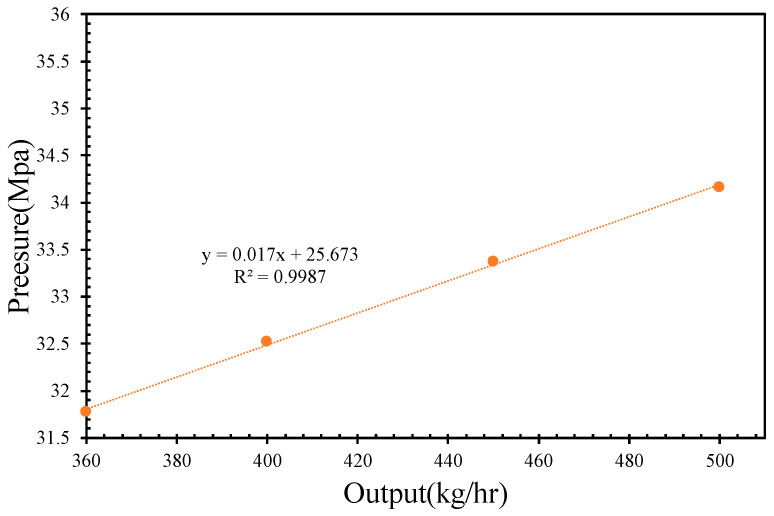
The pressure of the inlet with respect to the output rate.

**Figure 12:**
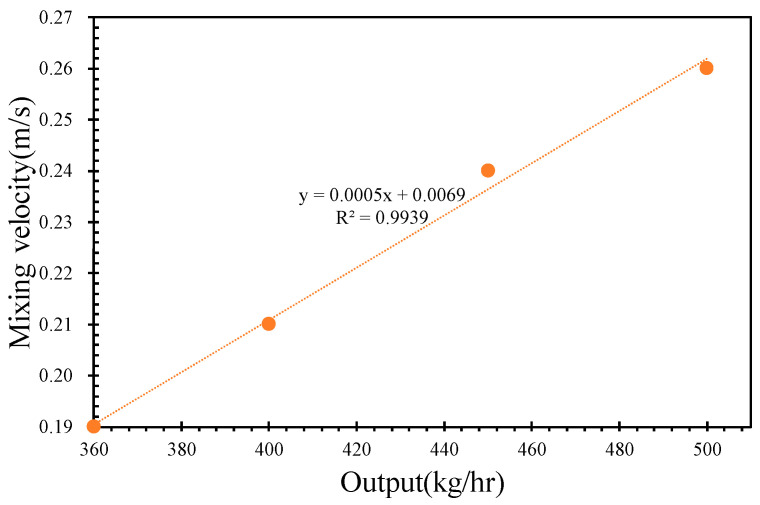
The velocity of the lip with respect to the output rate.

As the molten PVC flows from the screw/barrel system to the extrusion die, the pressure, temperature, and velocity distributions are numerically calculated using ANSYS Polyflow. Depicted for an inlet rate of 500 kg/h and a screw rotational speed of 43 rpm, the two-dimensional pressure distribution of molten PVC within the extrusion die is shown in Figure 10a. From the inlet to the outlet of the extrusion die, the pressure of the molten material decreases, which facilitates an increase in the flow velocity, as illustrated in Figure 10b. Consequently, the sectional area of the flow channel within the extrusion die gradually reduces, while the flow velocity increases to 0.04 m/s. A regional view of the die lip, shown in Figure 10c, reveals a significant decrease in pressure and an increase in the molten velocity at the die lip. Figure 11 demonstrates a linear correlation between the inlet pressure and the outlet flow rate of the extrusion die, with the inlet pressure increasing in response to higher outlet rates. Figure 12 further illustrates that the extrusion velocity at the die lip increases as the outlet rate increases. The extrusion velocity at the die lip ranges from 0.19 to 0.26 m/s as the outlet rate increases from 360 to 500 kg/h.

**Figure 13:**
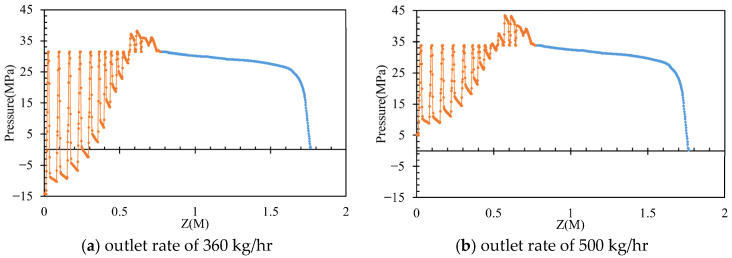
The pressure connection of molten PVC in the compound and extrusion.

A quantitative evaluation of screw compounding and extrusion die performance is demonstrated in Figure 7, Figure 9 and Figure 11. In Figure 12, the extrusion velocity at the die lip is 0.19 m/s at an outlet rate of 360 kg/h. The corresponding inlet pressure of the extrusion die at this outlet rate is 31.8 MPa, as shown in Figure 11. At a screw rotational speed of 32 rpm, the pumping pressure of molten PVC is 32 MPa, as depicted in Figure 9. By connecting the pressure of the molten PVC in the metering section to the extrusion die pressure, a pressure drop of −15 MPa at the entrance of the metering section is derived to facilitate the suction of molten PVC, as illustrated in Figure 13a. At an inlet rate of 500 kg/h, the inlet pressure of molten PVC for the extrusion die is 34 MPa. However, as shown in Figure 13b, the pumping pressure in Figure 6 is only 29 MPa, which is less than the required pressure of 34 MPa. To achieve optimal pressure alignment between the compounding screw and the extrusion die, the optimal outlet rate is found to be 439 kg/h at a screw rotational speed of 43 rpm.

## 4. Conclusions

The effects of various screw configurations including kneading, compressing, and mixing sections were examined for connection of the pumping pressure of the molten polymer by the screw and the pressure drop of molten polymer in extrusion die by ANSYS Polyflow in twin-screw compounding and die extrusion for PVC extrusion. Results show a valuable insights into melt flow behaviors, offering more accurate and reliable predictions compared to traditional trial-and-error approaches for effectively balancing throughput, thermal stability, and pressure generation. By examining the effects of various screw configurations, including kneading, compressing, and mixing sections, this study identifies optimal conditions for PVC extrusion that prevent thermal degradation and ensure efficient processing. The influence of screw speed and inlet flow rate on pressure and temperature distributions within the screw and extrusion die is crucial for maintaining stable melt conditions and product quality. The optimal outlet rate of 439 kg/h, achieved with a screw speed of 43 rpm, ensures a balance between melt pressure, velocity, and temperature, minimizing the risk of degradation. Overall, the numerical approach outlined here enhances our understanding of polymer extrusion dynamics, providing a robust framework for designing more efficient and reliable extrusion systems for thermally sensitive polymers. This study lays the foundation for further optimization of the extrusion of materials like PVC, supporting the development of more sustainable and cost-effective production processes.

## Figures and Tables

**Table 1:** Material properties of PVC.

Solid Density [g/cm^3^]	1.367
Thermal conductivity [W/(m°C)]	0.0625
Specific heat [J/g°C]	0.6–1.25

**Table 2:** Compounding parameters of PVC.

Molten Temperature [°C]	200
Inlet rate [kg/h]	360–500
Outlet rate [kg/h]	360–500
Rotating speed of screw [rpm]	43
Barrel temperature [°C]	
Kneading configuration	200
Compressing configuration	160
Mixing configuration	160
Die temperature [°C]	200

## Data Availability

All the data generated or analyzed during this study are included in the published article.
